# Supplementary material for: Content-rich biological network constructed by mining PubMed abstracts
Source: BMC Bioinformatics. 2004 Oct 8;5:147. doi: 10.1186/1471-2105-5-147 (PMC528731; doi:10.1186/1471-2105-5-147)
Supplement: Additional File 2 — The original results of the above study (non-essential files are deleted to keep the file size under the limit set by BMC bioinformatics). [file 1471-2105-5-147-S2.bz2 › chilibotAdditionalFile2/dip05/708ID11777918E18045/html/GYRA_GYRI.html]

 


 **GYRA** and **GYRI** 
  
Found 1 abstracts in PubMed, retrieved 1.  
 

 What does Google say? 
 PDF only 
| .edu only 

---

**Interactive relationship** (e.g. stimulation, inhibition, etc)

**Neutral relationship**- Second, surface plasmon resonance experiments indicated that  **GyrI**  binds to the gyrase holoenzyme with higher affinity than to either the  **GyrA**  or GyrB subunit alone.  Ref: 11777918 J Biol Chem, 2002

**Non-interactive relationship** (e.g. studied together, co-existance, homology, etc.)

- First, immunoprecipitation experiments revealed that  **GyrI**  interacts preferentially with the holoenzyme in an ATP independent manner, although a weak interaction was also detected between  **GyrI**  and the individual  **GyrA**  and GyrB subunits.  Ref: 11777918 J Biol Chem, 2002
